# Hepatoprotective Effect of Pretreatment with *Thymus vulgaris* Essential Oil in Experimental Model of Acetaminophen-Induced Injury

**DOI:** 10.1155/2014/954136

**Published:** 2014-02-04

**Authors:** Renata Grespan, Rafael Pazinatto Aguiar, Frederico Nunes Giubilei, Rafael Rocco Fuso, Marcio José Damião, Expedito Leite Silva, Jane Graton Mikcha, Luzmarina Hernandes, Ciomar Bersani Amado, Roberto Kenji Nakamura Cuman

**Affiliations:** ^1^Department of Pharmacology and Therapeutics, State University of Maringá, 87020-900 Maringá, PR, Brazil; ^2^Department of Chemistry, State University of Maringá, 87020-900 Maringá, PR, Brazil; ^3^Department of Clinical Analysis, State University of Maringá, 87020-900 Maringá, PR, Brazil; ^4^Department of Morphophysiology Sciences, State University of Maringá, 87020-900 Maringá, PR, Brazil

## Abstract

Acute liver damage caused by acetaminophen overdose is a significant clinical problem and could benefit from new therapeutic strategies. *Objective*. This study investigated the hepatoprotective effect of *Thymus vulgaris* essential oil (TEO), which is used popularly for various beneficial effects, such as its antiseptic, carminative, and antimicrobial effects. The hepatoprotective activity of TEO was determined by assessing serum aspartate aminotransferase (AST), alanine aminotransferase (ALT), and alkaline phosphatase (ALP) in mice. Their livers were then used to determine myeloperoxidase (MPO) enzyme activity and subjected to histological analysis. In vitro antioxidant activity was evaluated by assessing the free radical 2,2-diphenyl-1-picrylhydrazyl (DPPH•)-scavenging effects of TEO and TEO-induced lipid peroxidation. TEO reduced the levels of the serum marker enzymes AST, ALT, and ALP and MPO activity. The histopathological analysis indicated that TEO prevented acetaminophen-induced necrosis. The essential oil also exhibited antioxidant activity, reflected by its DPPH radical-scavenging effects and in the lipid peroxidation assay. These results suggest that TEO has hepatoprotective effects on acetaminophen-induced hepatic damage in mice.

## 1. Introduction 

Acetaminophen (APAP) at large doses causes serious liver injury that may develop into liver failure [1]. Hepatotoxicity induced by acetaminophen occurs through a biotransformation reaction that forms the reactive metabolite *N*-acetyl-p-benzoquinone imine (NAPQI) through the cytochrome P-450 mixed function of oxidase system. The metabolite is normally detoxified through a conjugation reaction with reduced glutathione (GSH). However, at large doses of acetaminophen, NAPQI levels increase, ultimately depleting GSH levels. Subsequently, sulfhydryl groups of hepatic proteins may react with the reactive metabolite, resulting in hepatic necrosis [2, 3]. Hepatocellular degeneration and necrosis are also associated with elevated enzyme markers, such as serum alanine aminotransferase (ALT) and aspartate aminotransferase (AST) that indicate hepatotoxicity [4]. Liver injury induced by acetaminophen in mice is a commonly used experimental model for screening substances with potential hepatoprotective activity [5]. Growing interest has been observed in the analysis of these natural entities for their potential benefits to human health. Accelerating research of plants used in folk medicine to treat liver diseases and boost liver function has been performed. In plants, essential oils are natural mixtures of terpenes, mainly monoterpenes and sesquiterpenes, which have been increasingly used in complementary therapies because essential oils are usually rich sources of phytochemical mixtures [6].

Essential oils extracted from fresh leaves and flowers can be used as aroma additives in food, pharmaceuticals, and cosmetics [7]. The leaves of thyme (*Thymus vulgaris*) can be used fresh or dried as a spice. Thyme also possesses various beneficial effects, including antiseptic, carminative, antimicrobial, and antioxidative effects [8]. Recently, in our laboratory it was showed that constituents, thymol and carvacrol, of *Thymus vulgaris *L. essential oil present effects on the inflammatory response [9]. Besides, essential oils and phenolic compounds, such as thymol in thyme, have antioxidative properties and may have hepatoprotective properties [8, 10]. To our knowledge, scarce information is available about the effects of *Thymus vulgaris *essential oil (TEO) in experimental hepatotoxicity models. Therefore, the present study investigated the hepatoprotective effect of TEO on acetaminophen-induced hepatic damage in mice.

## 2. Methods 

### 2.1. Extraction of Essential Oil

The fresh leaves of *Thymus vulgaris *L. were collected from the Proffessor Irenice Silva Medicinal Plant Garden on the campus of the State University of Maringá, Paraná, Brazil. The leaves were identified and authenticated by botanist Maria Aparecida Sert. A voucher specimen was deposited in the Herbarium of the Department of Botany, State University of Maringá (number 11329). The essential oil was obtained by hydrodistillation using a Clevenger-type apparatus. Approximately 556 g of the leaves was subjected to steam distillation for 2 h. The oil was dried over sodium sulfate and stored in an amber flask at 4°C. The TEO yield was 1.76% v/w.

### 2.2. Essential Oil Analysis

#### 2.2.1. Gas Chromatography-Mass Spectrometry (GC-MS)

Gas chromatographic (GC) analysis was performed with a Thermo Electron Corporation, Focus GC model, under the following conditions: DB-5 capillary column (30 m × 0.32 mm, 0.50 mm); column temperature, 60°C (1 min) to 180°C at 3°C/min; injector temperature 220°C; detector temperature 220°C; split ratio 1 : 10; carrier gas He; flow rate: 1.0 mL/min. The volume injected 1 *μ*L diluted in chloroform (1 : 10). The GC/MS analysis was performed in a Quadrupole mass spectrometer (Thermo Electron Corporation, DSQ II model), operating at 70 V. Identification of the individual components was based on comparison of their GC retention indices (RI) on apolar columns and comparison with mass spectra of authentic standard purchased from Sigma-Aldrich literature data.

#### 2.2.2. Nuclear Magnetic Resonance (NMR)

^1^H (300.06 MHz) and ^13^C NMR (75.45 MHz) spectra were recorded in deuterated chloroform (CDCl_3_) solution in a Mercury-300BB spectrometer, with *δ* (ppm) and spectra referred to CDCl_3_ (*δ* 7.27 for ^1^H and 77.00 for ^13^C) as internal standard.

### 2.3. Animals

Male Balb/c mice, weighing 24 ± 2 g, were provided by the Central Animal House of the State University of Maringá. The animals were housed at 22 ± 2°C under a 12/12 h light/dark cycle. Prior to the experiments, the animals fasted overnight, with water provided *ad libitum*. The experimental protocols were approved by the Ethical Committee in Animal Experimentation of the State University of Maringá (CEAE/UEM 126/2010).

### 2.4. Treatment of Animals

The experimental animals were divided into six groups of five animals each. Firstly, each group received orally during seven days the following treatment: Group I, the mice did not receive any treatment. In Group II, the mice received TEO vehicle (saline that contained 0.1% Tween 80). In Groups III–V, the mice were pretreated with TEO at doses of 125, 250, and 500 mg/kg, respectively. In Group VI, the mice were pretreated with the standard drug, silymarin (200 mg/kg). After this time, the animals fasted for 8 h and then received oral acetaminophen on the seventh day at a dose of 250 mg/kg in Groups II–VI. Group I orally received saline that contained 0.1% Tween 80 (APAP vehicle). After 12 h, the mice were anesthetized with halothane, and blood was collected for the determination of serum AST, ALT, and alkaline phosphatase (ALP). The livers were then used to determine myeloperoxidase (MPO) enzyme activity and for histological analysis.

### 2.5. Determination of Serum ALT, AST, and ALP Levels

Blood samples were collected and centrifuged at 3000 ×g for 15 min at 4°C. Serum ALT, AST, and ALP levels were then measured using the Analyze Gold enzymatic test kit.

### 2.6. Determination of MPO Activity

The livers were used to determine MPO enzyme activity in the homogenate supernatant of the liver sections, which were placed in potassium phosphate buffer that contained hexadecyltrimethylammonium bromide in a Potter homogenizer. The homogenate was stirred in a vortex and centrifuged. Ten microliters of the supernatant was added to each well in triplicate in a 96-well microplate. Two hundred microliters of the buffer solution that contained 16.7 mg *O*-dianisidine dihydrochloride (Sigma), 90 mL double-distilled water, 10 mL potassium phosphate buffer, and 50 *μ*L of 1% H_2_O_2_ was added. The enzymatic reaction was stopped by the addition of sodium acetate. Enzyme activity was determined by absorbance measured at 460 nm using a Spectra Max Plus microplate spectrophotometer.

### 2.7. Histopathological Analysis

The livers were washed in 0.9% (w/v) sodium chloride solution and placed in 10% neutral buffered formalin for fixation. Subsequently, the livers were processed to paraffin embedded and sectioned in semiserial at a 6 *μ*m thickness on a Leica rotary microtome (Leica Microsystems, Gladesville, New South Wales, Australia). The sections were stained with hematoxylin and eosin to evaluate tissue morphology using light microscopy (Olympus BX-41, Tokyo, Japan). The graded lesions were subjectively classified as absent, mild, moderate, or severe according to lesion area.

### 2.8. Lipid Peroxidation Assay

A lipid peroxidation assay was performed as previously reported with a minor modification [11]. Egg yolk homogenates were prepared as lipid-rich media. Briefly, 0.1 mL of TEO (5, 50, 500, 2500, and 5000 *μ*g/mL) in methanol was thoroughly mixed with 0.5 mL of egg yolk homogenate (10%, v/v, diluted with pure water) and made up to 1 mL with pure water. Ferrous sulfate (50 *μ*L, 70 mM) was added to induce lipid peroxidation, and the mixture was incubated for 30 min at 37.5°C. Afterward, 1.5 mL of 20% acetic acid (v/v, pH 3.5, diluted with pure water) and 1.5 mL of 0.8% (w/v) thiobarbituric acid in 1.1% sodium dodecyl sulfate (w/v, diluted with pure water) were added, and the resulting mixture was vortexed and heated at 95°C for 60 min. After cooling, 5 mL of 1-butanol was added to each tube and centrifuged at 5000 rotations per minute for 15 min. The organic upper layer was collected and measured spectrophotometrically at 532 nm using a Beckman DU-65 spectrophotometer. The essential oil was diluted in methanol (the solvent expressed no antioxidant activity). Ascorbic acid was used as a positive control. The inhibition of lipid peroxidation was calculated as follows: Inhibition  (%) = (1 − *A*_sample_/*A*_control_) × 100.  *A*_control_ was considered the absorbance of the control (i.e., methanol, instead of the sample). The IC_50_ value, representing the concentration of the essential oil that caused 50% inhibition of lipid peroxidation in the Fe^2+^/ascorbate system, was determined by linear regression analysis from the obtained inhibition (%) values.

### 2.9. DPPH Assay

Free radical-scavenging capacity (RSC) was evaluated by measuring the 2,2-diphenyl-1-picrylhydrazil (DPPH)-scavenging activity of TEO. The DPPH assay was performed as previously described [12], with minor modifications. The samples (60–2500 *μ*g/mL) in methanol were mixed with 1 mL of a 25 mM DPPH• solution (Sigma, St. Louis, MO, USA), with the addition of 95% methanol to a final volume of 4 mL. The absorbance of the resulting solutions and blank (i.e., with the same chemicals, with the exception of the sample) were recorded against ascorbic acid (Chem Cruz; used as a positive control) after 30 min at room temperature. For each sample, four replicates were recorded. The disappearance of DPPH• was measured spectrophotometrically at 515 nm using a Beckman DU-65 spectrophotometer. The percentage of RSC was calculated using the following equation: RSC (%) = 100 × (*A*_blank_ − *A*_sample_/*A*_blank_). The IC_50_ value, representing the concentration of the essential oil that caused 50% RSC inhibition, was determined by linear regression analysis from the obtained RSC values.

## 3. Results 

The thyme essential oil showed a predominance of carvacrol (45.54%), *α*-terpineol (22.96%), and endo-borneol (14.29%) as the major components (data not shown). In the acute toxicological study, TEO tested orally showed an LD50 value of 4.000 mg/kg. All doses used in the present study were lowest of LD50 values observed. Consequently, no apparent behavioural side effects were observed in the animals during our studies. The high LD50 values also suggest that the TEO was relatively safe and nontoxic to the animals.

We evaluated the effects of TEO on serum enzyme markers. As shown in Table 1, acetaminophen-induced hepatic damage markedly elevated serum ALT, AST, and ALP enzyme levels compared with the normal animals. Pretreatment with 250 and 500 mg/kg TEO but not 125 mg/kg TEO for 7 days prior to acetaminophen administration markedly reduced serum ALT, AST, and ALP levels compared with vehicle-treated controls. The effect of TEO was also comparable to silymarin, a standard hepatoprotective agent.

The activity of MPO in TEO-pretreated mice that received doses of 250 and 500 mg/kg was significantly decreased (0.073 ± 0.008 and 0.069 ± 0.008 IU/L, resp.) compared with the group that received acetaminophen only (0.251 ± 0.149 IU/L; Table 1).

**Figure 1:**
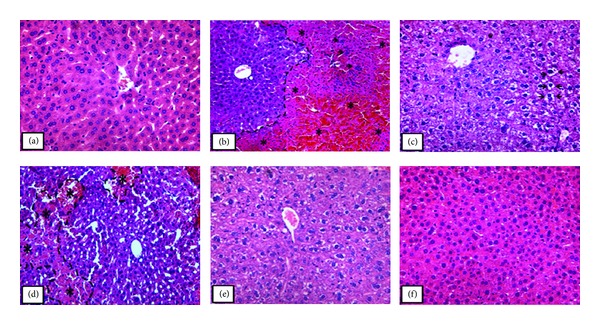
Photomicrograph of the liver in mice that received orally (a) saline, (b) acetaminophen on last day of treatment (250 mg/kg), (c) silymarin (200 mg/kg), and ((d)–(f)) acetaminophen, after being treated for 7 days with the essential oil of *Thymus vulgaris* (TEO), 125, 250, and 500 mg/kg, respectively. In (a) the liver showed normal morphology; (b) presence of necrosis and hemorrhagic points (*) in the defined area; ((c) and (e)) parenchyma stands out for having vacuolated hepatocytes (arrows); (d) observed necrotic areas (*); (f) hepatic parenchyma morphology similar to that observed in the control. Original magnification 40x in (a), (c), (e), and (f); original magnification 20x in (b) and (d). The sections stained with hematoxylin and eosin.

The histopathological analysis of control group (APAP vehicle) did not show hepatocellular damage (Figure 1(a)). However, the acetaminophen-treated group showed severe injury characterized by hemorrhagic and necrotic areas, presence of inflammatory infiltrate and piknotic nucleus, (Figure 1(b)). Considering the sylimarin group (standard drug), although the hepatic parenchymal did not present homogenous, necrotic areas were not observed. Also, cellular nucleus was more basophilic than of that control mice and many cells showed cytoplasm vacuolization, showing minor injuries (Figure 1(c)). The group of animals treated with TEO 125 mg/Kg showed interspersed necrotic areas with non-necrotic areas, with cytoplasm vacuolization and hemorrhagic points, characteristics of moderate injuries (Figure 1(d)), differently to that observed after TEO 250 mg/kg treatment, where mild injuries were observed, characterized by basophilic nucleus almost piknotic with shrinkage level (Figure 1(e)). Besides, the group treated with 500 mg/kg of TEO showed the hepatic parenchyma with similar morphology to the control group (Figure 1(f)). Therefore, TEO appeared to provide significant protection against hepatocyte injury.

**Figure 2:**
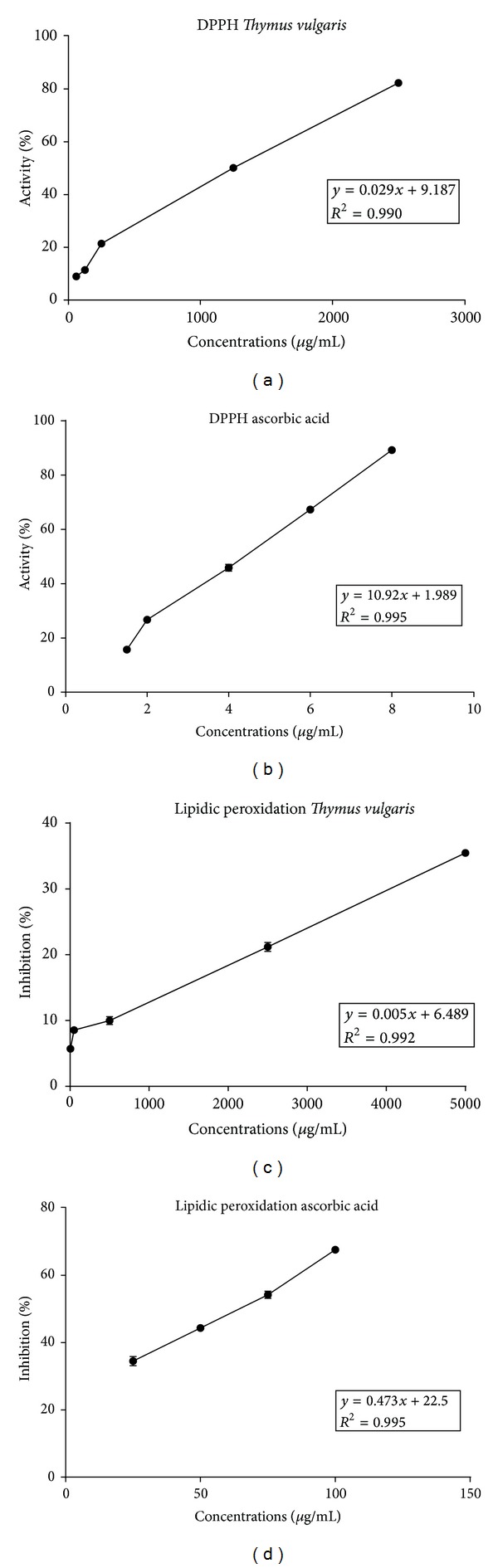
Antioxidant activity of the essential oil from *Thymus vulgaris.* The figure shows the percentage of neutralization of DPPH by (a) the essential oil of *T. vulgaris* and (b) ascorbic acid in the DPPH assay (µg/mL). The inhibition of lipid peroxidation (LP) in the Fe^2+^/ascorbate system induced by (c) the essential oil of *T. vulgaris *and (d) ascorbic acid in the TBA assay is also shown.

In the DPPH test, the ability of TEO to act as a donor for hydrogen atoms or electrons in the transformation of DPPH• in its reduced form (DPPH-H) was measured spectrophotometrically. The RSC of TEO at concentrations of 60–2500 *μ*g/mL showed significant antioxidant activity in vitro (IC_50_ = 1377 ± 1.6970 *μ*g/mL; Figure 2(a), Table 2). The IC_50_ value of ascorbic acid (i.e., the positive control) was 4.40 ± 0.07928 *μ*g/mL in the DPPH assay (Figure 2(b) and Table 2).

Egg yolk lipids undergo rapid lipid peroxidation when incubated in the presence of ferrous sulfate. The effect of TEO on nonenzymatic peroxidation is shown in Figure 2(c) and Table 2. At concentrations of 5–5000 *μ*g/mL, TEO significantly inhibited lipid peroxidation (IC_50_ = 8461 ± 7.778 *μ*g/mL). The IC_50_ value of ascorbic acid (i.e., the positive control) was 63.00 ± 3.3870 *μ*g/mL in the lipid peroxidation assay (Figure 2(d) and Table 2). Therefore, TEO was significantly correlated with total antioxidant activity (*R* > 0.99), demonstrating that TEO had antioxidant activity.

## 4. Discussion

In the present study, it was evaluated the hepatoprotective effect of TEO using the hepatotoxicity model induced by acetaminophen. This drug in an overdose (i.e., at doses that are different from analgesic doses that are safely and effectively used therapeutically) can induce severe hepatotoxicity in experimental animals and humans [13–15]. In our work, hepatotoxicity was reflected by a marked elevation of the levels of serum marker enzymes (AST, ALT, and ALP), increased MPO activity, and histopathologic alterations. These enzymes in serum are useful quantitative markers of the extent and type of hepatocellular damage. High levels of AST indicate a loss of the functional integrity of the liver, similar to the effects seen in viral hepatitis, cardiac infraction, and muscle injury. The ALT enzyme catalyzes the conversion of alanine to pyruvate and glutamate and is released in a similar manner. Therefore, ALT is more specific to the liver and thus is a better parameter for detecting liver injury [16–18].

Acetaminophen is converted to a toxic reactive intermediate called N-acetyl-p-benzoquinone imine (NAPQI) following metabolism by number of isozymes of cytochrome P-450 (CYPs), that is, CYP 2E1 [19], CYP 1A2 [20], CYP 2A6 [21], CYP 3A4, and CYP 2D6 [22]. NAPQI could be bound covalently to cellular proteins, including mitochondrial proteins [23] and that in turn leads to mitochondrial dysfunction. Mitochondrial respiration is inhibited, which results in the formation of reactive oxygen species (ROS) and peroxynitrite in the mitochondria [24, 25]. The massive production of reactive species (ROS) may lead to depletion of protective physiological moieties (glutathione and *α*-tocopherol), ensuing widespread propagation of the alkylation as well as peroxidation, causing damage to the macromolecules in vital biomembranes [26]. However, reduced glutathione is one of the main defense mechanisms against oxidative stress reducing peroxides and hydroperoxides [27]. In addition, the oxidative stress can induce a mitochondrial membrane permeability transition and adenosine-5′-triphosphate (ATP) depletion which results in membrane permeabilization, membrane rupture, and cell apoptosis [28–31].

We assessed the hepatoprotective effect of TEO in acetaminophen-induced hepatic damage in mice, and the results suggested that pretreatment with TEO could be protecting the functional integrity of hepatocytes and the cellular membrane from damage by toxic reactive metabolites produced by acetaminophen biotransformation [32, 33].

Inflammation also plays a central role during drug-induced acute hepatitis and products of arachidonic acid metabolism have been extensively involved in inflammatory processes [34]. The histopathological analysis of the livers obtained from the TEO-pretreated group showed mild sinusoidal congestion, less inflammatory cell infiltration, and well-preserved hepatocytes with less of an area of necrosis compared with the severe centrilobular necrosis observed in acetaminophen-treated mice. These results suggest that the anti-inflammatory properties of TEO are partially involved in the hepatoprotective effect of this essential oil. Similarly, previous studies have shown that extracts of plants protect the liver from acetaminophen overdose, suggesting that the hepatoprotective effect can be considered an expression of the functional improvement of hepatocytes that results from accelerated cellular regeneration [35, 36]. Thus, the cytoprotective effects of silymarin, a natural product, are also mainly attributable to its antioxidant and free radical-scavenging properties. Silymarin can interact directly with cell membrane components to prevent abnormalities in the content of the lipid fraction that is responsible for maintaining normal fluidity [37].

Furthermore, the free radical-initiated oxidation of cellular membrane lipids can lead to cellular necrosis and is now accepted to be important in various pathological conditions. High acetaminophen doses significantly elevated reactive oxygen species levels, ultimately depleting the levels of superoxide dismutase (SOD) and GSH in liver tissue. This oxidative stress contributes to the initiation and progression of liver damage [33]. Apoptosis is a form of cell death and has deleterious consequences as observed in many diseases, including acquired immunodeficiency syndrome, cancer, and neurodegenerative disorders [38–40]. Apoptosis is induced by different stimuli such as oxidants, xenobiotics, glucocorticoids, and irradiation [41] which converge to trigger a common pathway of cell death, activating proteases as caspase-3 found only in cells undergoing apoptosis. Protease caspase-3 can cleave and inactivate a nuclear protein poly (ADP-ribose) polymerase (PARP), an enzyme used for DNA repair. Many bioactive substances exert their effect on apoptosis acting in cell cycle progression and/or triggering apoptotic cell death. However, the effects of essential oils inducing or inhibiting apoptosis via mitochondrial stress and caspase activation are controversial [42–45]. Thus, various essential oils have been shown to have antioxidant activity and have been used as antioxidant drugs in many diseases [46–48]. The ability of natural products to reduce acetaminophen-induced lipid peroxidation could be as a result of their antioxidant constituents [49]. The mechanisms of TEO in reduction of free radical species and their effects on cells and tissues damage should be elucidated.

## 5. Conclusion 

TEO pretreatment improves the hepatotoxicity induced by acetaminophen in mice. The effects of TEO partially involve the antioxidative effect of this essential oil. However, further detailed studies are required to investigate the mechanism by which TEO exerts its effects and determine the specific constituents that are responsible for this action.

## Figures and Tables

**Table 1:** The effect of Thymus essential oil on biomarkers of hepatic damage.

Groups and designe of treatment	ALT	AST	ALP	MPO
	(IU L^−1^)			
Group I, control, Tween 80	48.88 ± 2.05	113.3 ± 15.39	138.40 ± 10.67	0.058 ± 0.009
Group II, APAP control (250 mg kg^−1^)	11130 ± 973.40^a^	6860 ± 140.00^a^	181.90 ± 24.75^a^	0.251 ± 0.149*ª*
Group III, 125 mg kg^−1^ of TEO + APAP	3847 ± 3673	3201 ± 2731	110.40 ± 35.74	0.101 ± 0.008
Group IV, 250 mg kg^−1^ of TEO + APAP	261.10 ± 84.41^b^	176.0 ± 76.50^b^	71.37 ± 11.49^b^	0.073 ± 0.009^b^
Group V, 500 mg kg^−1^ of TEO + APAP	110.70 ± 35.79^b^	110.8 ± 22.53^b^	79.20 ± 1.591^b^	0.069 ± 0.009^b^
Group VI, 200 mg kg^−1^ of SLM + APAP	388.80 ± 148.10^b^	286.7 ± 150.40^b^	101.40 ± 40.01^b^	0.091 ± 0.003^b^

Values are mean ± SEM. 5 mice in each group (*n* = 5), *P* < 0.05 values are considered statistically significant. ^a^ *P* < 0.05 acetaminophen (APAP) treated group compared with animals in control groups. ^b^ *P* < 0.05 mice treated with the Thyme essential oil (TEO) or Silymarin (SLM) compared with acetaminophen group.

**Table 2:** Summary of IC_50_ values of thyme essential oil (TEO) and ascorbic acid.

	DPPH IC_50_ (*μ*g/mL) ± SD	Lipid peroxidation IC_50_ (*μ*g/mL) ± SD
TEO	1377 ± 1.6970	8461 ± 7.7781
Ascorbic acid	4.40 ± 0.07928	63.00 ± 3.3870
